# Social, dietary and clinical correlates of oedema in children with severe acute malnutrition: a cross-sectional study

**DOI:** 10.1186/s12887-015-0341-8

**Published:** 2015-03-22

**Authors:** Maren Johanne Heilskov Rytter, Hanifa Namusoke, Esther Babirekere-Iriso, Pernille Kæstel, Tsinuel Girma, Vibeke Brix Christensen, Kim F Michaelsen, Henrik Friis

**Affiliations:** Department of Paediatrics, Copenhagen University Hospital, Rigshospitalet, Blegdamsvej 9, 2100 Copenhagen, Denmark; Mwanamugimu Nutrition Unit, Department of Paediatrics, Mulago Hospital, Kampala, Uganda; Department of Nutrition, Exercise and Sports, University of Copenhagen, Rolighedsvej 30, 1958 Frederiksberg C, Denmark; Department of Paediatrics and Child Health, Jimma University Specialized Hospital, Jimma, Ethiopia

**Keywords:** Malnutrition, Kwashiorkor, Marasmus, Oedema, Breastfeeding, Diet, Infection, Acute phase proteins, Maternal deprivation

## Abstract

**Background:**

Severe acute malnutrition is a serious public health problem, and a challenge to clinicians. Why some children with malnutrition develop oedema (kwashiorkor) is not well understood. The objective of this study was to investigate socio-demographic, dietary and clinical correlates of oedema, in children hospitalised with severe acute malnutrition.

**Methods:**

We recruited children with severe acute malnutrition admitted to Mulago Hospital, Uganda. Data was collected using questionnaires, clinical examination and measurement of blood haemoglobin, plasma c-reactive protein and α_1_-acid glycoprotein. Correlates of oedema were identified using multiple logistic regression analysis.

**Results:**

Of 120 children included, 77 (64%) presented with oedematous malnutrition. Oedematous children were slightly older (17.7 vs. 15.0 months, p = 0.006). After adjustment for age and sex, oedematous children were less likely to be breastfed (odds ratio (OR): 0.19, 95%-confidence interval (CI): 0.06; 0.59), to be HIV-infected (OR: 0.10, CI: 0.03; 0.41), to report cough (OR: 0.33, CI: 0.13; 0.82) and fever (OR: 0.22, CI: 0.09; 0.51), and to have axillary temperature > 37.5°C (OR: 0.28 CI: 0.11; 0.68). Household dietary diversity score was lower in children with oedema (OR: 0.58, CI: 0.40; 85). No association was found with plasma levels of acute phase proteins, household food insecurity or birth weight.

**Conclusion:**

Children with oedematous malnutrition were less likely to be breastfed, less likely to have HIV infection and had fewer symptoms of other infections. Dietary diversity was lower in households of children who presented with oedema. Future research may confirm whether a causal relationship exists between these factors and nutritional oedema.

**Electronic supplementary material:**

The online version of this article (doi:10.1186/s12887-015-0341-8) contains supplementary material, which is available to authorized users.

## Background

Severe acute malnutrition (SAM) in children is a serious public health problem [1], and a challenge for clinicians [2]. SAM manifests as non-oedematous or oedematous malnutrition [3]. Oedematous malnutrition is an enigmatic and life-threatening condition, covering a clinical spectrum from simple nutritional oedema to fulminant kwashiorkor, with anaemia, hepatomegaly, mental changes and dermatosis, in addition to oedema. WHO currently define oedematous malnutrition by bilateral pitting oedema, regardless of whether other symptoms of kwashiorkor are present [4].

Although oedematous malnutrition was first described in scientific literature more than 80 years ago [5], the pathophysiology of the syndrome, and the mechanism behind the formation of oedema remains unknown [6]. Various explanations have been suggested: protein deficiency [7], hormonal imbalance [8], aflatoxin poisoning [9], and oxidative stress [10], but none have been verified, and a large intervention trial failed to prevent oedematous malnutrition with antioxidant supplements [11]. A retrospective study from Jamaica found higher birth weights in children with oedematous than non-oedematous malnutrition, and suggested pre-natal factors to influence the clinical phenotype of malnutrition [12].

One way to generate hypotheses about the causes of oedematous malnutrition is to assess how children with this condition differ from children with non-oedematous malnutrition in terms of socio-demographic background, clinical characteristics, and household diet. However, few previous studies have investigated this, with conflicting results, and with few exceptions [13], most were carried out in the last century, when diagnostic criteria for malnutrition differed from those used today.

The objective of this study was therefore to present an up-to-date investigation of socio-demographic, dietary and clinical correlates of oedema, in children hospitalised with SAM in a nutrition unit in Uganda.

## Methods

### Study design

This cross-sectional study is based on baseline data from a cohort study of children admitted for in-hospital treatment of SAM, between October 2012 and February 2013.

### Study site and standard treatment

Mwanamugimu Nutrition Unit, Mulago Hospital, Uganda, is the main national rehabilitation centre for children with complicated SAM. Patients were treated following the Ugandan National Protocol for Integrated Management of Acute Malnutrition [14] using therapeutic diets, F75 and F100, and empiric parenteral antibiotics, followed by outpatient treatment with ready-to-use therapeutic food. At the time of the study, the outpatient clinic was only working one day per week, so referred children were not routinely assessed with appetite tests to determine whether they should receive in-patient or out-patient treatment. However, all admissions came through the hospitals acute care unit, meaning that all children had been evaluated sick enough to require hospital admission. All biological mothers were offered counselling and testing for HIV, following World Health Organization (WHO) guidelines [15]. If the mother was HIV-infected or absent, the child was tested.

### Inclusion and exclusion criteria

Children were eligible if they were admitted on weekdays for treatment of SAM, defined as either weight-for-height z-score < −3, using the WHO Growth Standard, or mid-upper arm circumference (MUAC) < 11.5 cm, or bilateral pedal pitting oedema. Children had to be 6–59 months old, live near the hospital, and their parent or guardian had to give informed consent. Children were excluded if they had significant disability (like cerebral palsy); shock or severe respiratory distress requiring resuscitation at admission; haemoglobin < 4 g/dl, or a body weight < 4.5 kg.

### Questionnaire

Caretakers were asked about the household where the child had lived during the two months preceding admission, about the child’s breast-feeding history and symptoms present. Caretakers were asked to state which one of their child’s symptoms they perceived as most severe. If the mother was present, she was asked to recall the child’s birth weight.

Dietary data was collected by asking about whether seven types of high-quality foods were served in the household during the last two weeks (Additional file 1: Figure S1). The foods were locally available, but likely in limited amounts in resource-poor households. From this, a simple dietary diversity score (DDS) was calculated, as the sum of different food types served.

Food insecurity was evaluated using the validated Household Food Insecurity Access Scale (HFIAS) [16]. The scale consists of nine questions regarding perceived food insecurity, each with four frequency options, from “never” to “more than ten times in the past four weeks”. A HFIAS score from 0–27 was calculated, and households classified as having no, mild, moderate, or severe food insecurity.

### Examinations

On admission, oedema was diagnosed according to guidelines [17], and axillary temperature was measured. Anthropometric measurements were done by one nurse, trained in anthropometry, assisted by the child’s caretaker. Measurements were done in triplicate, and the average of three measurements was used. Length was measured using an infant length board and MUAC using measuring tape, both to the nearest 1 mm. Body weight was measured daily to the nearest 100 g using a digital scale. Anthropometric z-scores were computed in Stata using the command “zscore06” based on the 2006 WHO Growth Standards [18]. The lowest weight recorded during admission was used to compute z-scores for all children, to use the weight free from oedema. Maternal weight was measured using a digital scale with a precision of 100 g and height measured with a precision of 1 mm using a wall-mounted stadiometer. We did not by default remove extreme anthropometric values, but it was checked that they were likely to be real, by being present on different days, and by being in accordance with other measurements (e.g. those with extreme z-scores also had very low MUAC). To assess appetite, it was noted whether the child consumed all of the first served F75, or not.

### Blood samples

On admission, haemoglobin was measured in venous blood collected in heparinized Vacutainer tubes, using HemoCue (Hb 201+, Ängelholm, Sweden). Plasma was obtained from a Vacutainer with citrate (Cell-Preparation Tube, Becton Dickinson, USA), stored at -80C°, until shipped to Denmark on dry ice, where plasma (P-) level of C-reactive protein (CRP) and α_1_-acid glycoprotein (AGP) was measured at University of Copenhagen, Department of Nutrition, Exercise and Sports, with ABX Pentra 400 (Horiba, France).

### Ethical issues

The study was approved by Makerere University School of Medicine’s Research Ethics Committee, and Uganda National Council of Science and Technology. A consultative approval was obtained from the Danish National Board of Research Ethics. Parents or guardians willing to participate signed a written informed consent form, after oral and written information in English and Luganda. Participation in the study did not affect the medical and nutritional treatment, which was similar to that given to admitted children not included in the study.

### Statistics

Data was double entered into EpiData (Odense, Denmark) and analysed using Stata 12 (StataCorp LP, College station, USA). To test differences in means between groups, t-tests were used for normally distributed variable, and for others (age, CRP, DDS and HFIAS score), Mann–Whitney rank-sum-tests were used. Chi-square tests were used to test for differences in proportions between groups. For analysis of CRP, children were classified as having CRP levels above or below 10 mg/L. The relationship between CRP and body temperature was examined with linear regression using the log-transformed CRP values. Correlates of oedema were identified using logistic regression, unadjusted and adjusted for sex and age in months, and results presented as odds ratios (OR) with 95% confidence intervals (CI). Additionally, background and clinical characteristics were analysed adjusted for age, sex, HIV-status, and breastfeeding status. Dietary factors and food insecurity were also analysed adjusting for age, sex, breastfeeding status and for month of inclusion, to account for seasonality.

## Results

**Figure 1:**
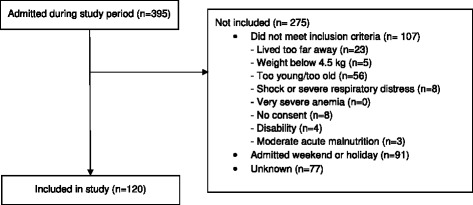
**Flow diagram for inclusion into the study.**

Of 395 patients admitted to the unit during the study period, 120 (30%) were included in the study (Figure 1). The reason for exclusion was known in 198 of non-included children, but unknown in 77 children. This could be due to insufficient completion of the screening log, or because some of these children were transferred from other hospital departments to MNU, and therefore missed screening. Among included children the median (range) age was 15.9 (6–53) months, and 62% were boys. Almost half of the children’s parents were not living together and 29 (25%) of the children had not stayed with their mother during the preceding two months (Table 1).

**Table 1:** **Background characteristics of 120 children admitted with severe acute malnutrition** ^**a**^

	**N** ^**b**^	
Female sex	120	38 (46)
Age, months	120	15.9 (12.6; 21.9)
Mothers education attended (not necessarily completed)	97	
No school		6 (6)
Primary school		43 (42)
Secondary school		43 (42)
More than secondary school		7 (7)
**Household characteristics**		
Urban or peri-urban location	103	85 (88)
Occupation, head of household	93	
Unemployed		14 (13)
Agriculture		9 (8)
Domestic service, unskilled manual		25 (23)
Skilled manual, sales and service		39 (36)
Professional/technical/management		14 (13)
Drinking water available on premises	107	20 (21)
Improved, not shared, toilet facility	94	18 (17)
Household owns agricultural land	109	36 (39)
Household owns cattle	105	6 (6)

^a^Values presented are % (N), or median (25%; 75%). ^b^N = number of children in whom information was available.

### Clinical correlates of oedema

Of the 120 included children, 77 (64%) presented with oedematous malnutrition (Table 2), of which 6 (8%) had grade 1 oedema (only feet), 22 (28%) had grade 2 oedema (up to the knees), and 49 (64%) had grade 3 oedema (above the knees). Oedematous children were slightly older than non-oedematous (17.7 vs. 15.0 months, p = 0.006). Seven percent of oedematous and 33% of non-oedematous children were still breastfed, and breastfeeding remained negatively associated with oedema, controlling for age and sex (OR: 0.19, CI: 0.06; 0.59).

**Table 2:** **Clinical and biochemical correlates of oedema among 120 children with severe acute malnutrition on hospital admission**

		**Oedema** ^**a**^	**No oedema** ^**a**^		**Odds Ratio (95% CI)** ^**b**^	
	**N** ^**c**^	**n = 77**	**n = 43**	**p**		**p**
Female sex	120	36 (28)	42 (18)	0.55	0.85 (0.38; 1.86)	0.68
Age, months	120	17.7 (13.5; 23.3)	15.0 (10.5; 19.5)	0.006	1.06 (1.01;1.12)	0.03
Currently breastfed	111	7 (5)	33 (14)	<0.001	0.19 (0.06; 0.59)	0.004
Lived with mother last two months	114	68 (49)	86 (36)	0.04	0.42 (0.15; 1.17)	0.10
**Anthropometric data**						
Mid-upper arm circumference, cm	117	12.1 ± 1.4	10.6 ± 0.6	<0.0001	3.72 (2.18; 6.35)	<0.001
Weight-for-height, Z^d^	120	−3.0 ± 1.4	−4.1 ± 1.0	<0.0001	2.10 (1.43; 3.08)	<0.001
Height-for-age, Z	120	−3.1 ± 1.4	−3.2 ± 1.5	0.58	1.47 (1.05; 2.06)	0.03
Recalled birth weight, kg^e^	88	3.4 ± 0.9	3.5 ± 0.9	0.53	0.84 (0.51; 1.39)	0.49
**Child’s clinical data**						
HIV-infected children	104	12 (8)	31 (12)	0.02	0.10 (0.03; 0.41)	0.001
Reported symptoms on admission						
Diarrhoea	112	43 (17)	63 (25)	0.04	0.45 (0.20; 1.02)	0.06
Cough	112	54 (39)	78 (31)	0.02	0.33 (0.13; 0.82)	0.02
Fever	112	26 (19)	65 (26)	<0.001	0.22 (0.09;0.51)	<0.001
Axillary temperature > 37.5°C	116	16 (12)	43 (18)	0.002	0.28 (0.11; 0.68)	0.005
Axillary temperature < 36.0°C	116	16 (12)	0 (0)	0.006	-^g^	-
Observed appetite^f^	95	83 (52)	69 (22)	0.13	2.55 (0.89; 7.31)	0.08
**Biochemical data on admission**						
Plasma c-reactive protein, mg/L	83	17.9 (6.2; 32.7)	20.0 (12.1; 42.3)	0.51	0.99 (0.98; 1.00)	0.28
> 10 mg/L		67 (38)	77 (20)	0.35	0.48 (0.16; 1.43)	0.19
Plasma α_1_-acid glycoprotein, g/L	83	2.46 ± 0.62	2.26 ± 0.92	0.26	1.35 (0.70; 2.60)	0.38
Haemoglobin, g/dL	112	8.9 ± 2.2	9.2 ± 2.5	0.46	0.98 (0.82; 1.17)	0.82

^a^Values presented are % (n), median (25%;75%) or mean ± SD; ^b^Odds ratio calculated by logistic regression, adjusted for age and sex; ^c^N = number of children for whom information is available; ^d^Z-scores were computed based on the lowest weight recorded (after loss of oedema), for all children; ^e^Only asked if mother was present; ^f^Noted if the child was able to consume all of the first serving of F75; ^g^Cannot be estimated since no non-oedematous children had temperature < 36.0°C.

More oedematous children had lived separated from their mother during the last two months (32% *vs*. 14%), but this difference was not significant when controlling for age and sex. There was no crude difference in height-for-age z-score (HAZ) between children with and without oedema. However, adjusting for age and sex, a positive association appeared between HAZ and oedema (OR: 1.47, CI: 1.05; 2.06). Children’s birth weights were recalled by 88 mothers: it was 3.4 kg in oedematous and 3.5 kg in non-oedematous children, and not significantly different.

Among children with oedematous malnutrition, 8 (12%) were HIV-infected, compared to 12 (31%) of non-oedematous children, and HIV-infection remained less frequent in oedematous children when adjusting for age and sex (OR: 0.10, CI: 0.03; 0.41). Diarrhoea, cough and fever were reported in fewer oedematous children, and axillary temperature >37.5°C was measured in 16% of oedematous children vs. 43% of non-oedematous children (p = 0.002). While no included children had hypothermia (axillary temperature < 35.5°C), sub-normal axillary temperature (<36.0°C) was seen in 12 (16%) of oedematous children, and in no non-oedematous children (p = 0.008). Although a positive association was seen between body temperature and CRP (β = 0.18, p = 0.04), the three children with the highest CRP values all had normal body temperature. We did not find any indications that the association between CRP and body temperature was different in oedematous and non-oedematous children. The symptom causing greatest worry to caretakers of oedematous children was “swelling” (61%), followed by “diarrhoea” (11%), while caretakers of non-oedematous children were most concerned about “diarrhoea” (29%), “weight loss” (24%), and “fever” (11%) (Table 3). Eighty-three percent of oedematous and 69% of non-oedematous children were able to consume all their first served F75, and thus, observed appetite was marginally better in oedematous children (OR: 2.55, CI: 0.89; 7.30).

**Table 3:** **The single symptom causing greatest worry to caretakers of oedematous and non-oedematous children, respectively** ^**a**^

	**Oedema**	**No oedema**	
	**n = 71**	**n = 38**	**p**
**Symptom**			<0.001
Swelling	61 (43)	8 (3)	
Diarrhoea	11 (8)	29 (11)	
Weight loss	4 (3)	24 (9)	
Fever	6 (4)	10 (4)	
Lack of appetite	3 (2)	10 (4)	
Vomiting	4 (3)	5 (2)	
Cough/difficulty breathing	3 (2)	8 (3)	
Rash	4 (3)	3 (1)	
Other	4 (3)	3 (1)	

^a^Data presented are % (n).

Sixty-seven percent of oedematous, and 77% of non-oedematous children had CRP > 10 mg/l, which was not significantly different, and neither were AGP or haemoglobin levels.

### Maternal data, household diet and food insecurity in association with oedema

Oedematous children came from larger households than non-oedematous children (Table 4). Of seven specific food types served in the household, nuts and fresh fruit were significantly less frequently served in households of oedematous children. There was also a trend for lower household consumption of fish and green leafy vegetables in oedematous children, although not significantly so. Total dietary diversity, obtained by adding up the number of different food types served, was negatively associated with oedema (OR: 0.58, CI: 0.40; 0.85), while food insecurity measured as HFIAS score was not.

**Table 4:** **Maternal and household data, household diet and food insecurity and their association with oedema among 120 children with severe acute malnutrition**

		**Oedema** ^**a**^	**No oedema** ^**a**^		**Odds Ratio (95% CI)** ^**b**^	
	**N** ^**c**^	**n = 77**	**n = 43**	**p**		**p**
**Maternal and household data**						
Mothers body-mass index, kg/m^2^	81	21.8 ± 2.9	21.3 ± 2.0	0.45	1.09 (0.91; 1.31)	0.36
Mother living with child’s father	116	52 (38)	56 (24)	0.70	0.88 (0.41; 1.92)	0.75
Mother pregnant or has younger child	102	22 (14)	8 (3)	0.08	2.83 (0.74; 10.8)	0.13
Number of people in household	113	5.6 ± 2.5	4.5 ± 1.7	0.008	1.33 (1.06; 1.67)	0.01
**Foods served in household last two weeks** ^**d**^						
Fish	100	55 (33)	73 (29)	0.08	0.42 (0.17; 1.02)	0.06
Nuts	97	83 (49)	95 (36)	0.09	0.17 (0.03; 0.88)	0.03
Eggs	98	51 (30)	49 (19)	0.84	1.22 (0.52; 2.88)	0.65
Meat	99	72 (43)	67 (26)	0.60	1.16 (0.46; 2.91)	0.75
Dairy products	97	66 (39)	82 (31)	0.10	0.38 (0.14; 1.08)	0.07
Green leafy vegetables	94	71 (40)	84 (32)	0.15	0.34 (0.11; 1.04)	0.06
Fresh fruits	98	73 (44)	87 (33)	0.11	0.26 (0.08; 0.87)	0.03
Dietary Diversity Score, 0–7^e^	90	5 (4; 6)	6 (5; 6)	0.02	0.58 (0.40; 0.85)	0.005
**Household Food Insecurity** ^**d**^						
Household Food Insecurity Access Scale Score, 0 -27	93	5.0 (0.0; 10.0)	8.5 (1.5; 15.5)	0.15	0.97 (0.91;1.03)	0.34
Food insecurity category	97			0.07		
Food secure		32 (19)	21 (8)		ref.	
Mildly food insecure		8 (5)	5 (2)		0.72 (0.10; 5.48)	0.75
Moderately food insecure		29 (17)	16 (6)		1.37 (0.37; 5.10)	0.64
Severely food insecure		31 (18)	58 (22)		0.35 (0.12; 1.04)	0.06

^a^Values presented are % (n), median (25%;75%) or mean ± SD; ^b^Odds ratio calculated by logistic regression, adjusted for age and sex; ^c^n = number of children for whom information is available; ^d^Only caretakers who had stayed with the child during the last two weeks were asked; ^e^Sum of different food types (listed above) served in household during last two weeks.

### Further adjusted analyses

In order to investigate whether any of the observed associations could be explained by HIV-infection or breastfeeding, we did further analyses adjusted for age, sex, HIV status and current breastfeeding (not shown in table). In this model, HIV-status and current breastfeeding were still negatively associated with oedema (HIV status: OR: 0.10, CI: 0.02; 0.50, breastfeeding OR: 0.24, CI: 0.02; 0.50), and age positively associated with oedema (OR 1.13, CI: 1.03; 1.23). Reported cough, diarrhoea and fever also remained negatively associated with oedema (cough: OR 0.13, CI: 0.03; 0.48, diarrhoea: OR: 0.32, CI: 0.11; 0.89, fever: OR: 0.15, CI: 0.05; 0.44), and so did elevated temperature measured on admission (OR 0.25, CI: 0.09; 0.73).

In contrast, appetite on admission was not associated with oedema when adjusting for age, sex, HIV-status and breastfeeding (OR: 2.13, CI: 0.56; 7.90), and neither was separation from the mother, household size, or having a pregnant mother or younger sibling.

We also analysed the data on household diet and food insecurity adjusting for age, sex, breastfeeding and month of the study, to account for seasonality. This did not change any of the observed associations.

## Discussion

Few studies have compared characteristics of children with nutritional oedema, to children with non-oedematous SAM. In our study, children with oedema were less likely to be breastfed, less likely to have HIV infection, reported fewer symptoms of infections and had higher HAZ, and the dietary diversity was lower in families of children who presented with oedema.

### Breastfeeding

Oedematous children were less likely to be breastfed, even after adjusting for age. Separation from the mother had more frequently occurred in children with oedematous malnutrition, and their mothers were more frequently pregnant or had given birth to a younger sibling, but both of these associations seemed mediated by lack of breastfeeding. The association with breastfeeding seemed to be independent of the association with HIV.

In Luganda, the main language spoken in Kampala, oedematous malnutrition is called *obwoosi* which means “the child has a pregnant mother”. Here, it is common practice to separate children from their pregnant mothers, causing abrupt weaning from breast milk (Esther Babirekere, personal communication, 2014). The term *kwashiorkor* was translated by Williams in 1935, as “the disease the disposed baby gets when the next is born”. She noted an association with an absent or sick mother, and lack of breastfeeding [19]. Later, other studies reported oedematous malnutrition to occur in breastfed children as well, leading the authors to question whether breastfeeding is indeed protective [20]. A study from Sudan found no difference in breastfeeding practice in children with different types of malnutrition [21], while studies from Lesotho [22] and Nigeria [23] found less breastfeeding in oedematous children, although no adjustments were made for the higher age of oedematous children.

Breastfeeding may protect against oedematous malnutrition by nutritional, immunological, microbial or social mechanisms. It is unknown whether certain nutrients are particularly deficient in oedematous malnutrition, but if so, it could be something usually provided in sufficient amounts in breast milk. Breast milk also contains immune modulating substances, and immunological mechanisms have been suggested to be involved in the pathogenesis of oedematous malnutrition [24]. Breastfed children have a different gut microbial flora from non-breastfed, which could also be a mechanism [25]. Finally, breastfeeding may be a marker of attachment between child and mother, and associated with maternal care and stimulation, and older studies have indeed hypothesized that emotional deprivation and abrupt breaking of the mother-infant bond could play a role in the development of oedematous malnutrition [26]. In our study, living away from the mother was no longer associated with oedema when breastfeeding was controlled for; however, disentangling the nutritional and social aspects of breastfeeding is difficult, and we cannot rule out that emotional or social factors are involved in a protective effect of breastfeeding against oedematous malnutrition.

### Infections

In agreement with other studies [24,27], we found that fewer oedematous children were HIV-infected. Oedematous children also had less caregiver-reported symptoms of infection, and less frequent measured temperature >37.5°C, associations that did not seem to be explained by the children’s HIV-status.

Malnutrition is often precipitated by infection [28], however, the pattern of infection in the two types of malnutrition has not received much attention. Our group recently reported more infections in non-oedematous children from a similar setting in Ethiopia [13]; more diarrhoea in non-oedematous children was reported from Kenya [29], while another Ugandan study found similar rates of bacteraemia in the two types of malnutrition [30]. These observations somewhat question the paradigm of oedematous malnutrition being particularly triggered by infections, which initiated the theory of oxidative stress causing oedematous malnutrition [10]. This theory has already been challenged by the results of a large randomized controlled trial which failed to prevent oedematous malnutrition in susceptible children with anti-oxidant supplements [11]. We also found that appetite was not significantly different and, if anything, poorer in non-oedematous children, also challenging the classical idea of oedematous malnutrition being particularly associated with anorexia, although the appetite recorded was simply whether or not the child completed its first feed of F75.

However, this pattern of infection could be explained by care-seeking behaviour of parents and clinicians: It seems plausible that a caretaker would seek help for a child with oedema, regardless of what other symptoms the child may have. In contrast, a parent may not necessarily seek help for a child who is thin, unless other symptoms develop. A hospital setting may therefore select non-oedematous children with co-infections, but not necessarily oedematous children with co-infections. This explanation is supported by answers to the question: “what symptom worries you most?”. Caretakers of children with oedema most frequently responded “body swelling”, while caretakers of non-oedematous most frequently responded “diarrhoea”. Plausibly, this pattern of infection could be different if malnourished children were identified by community screening.

In contrast to reported symptoms of infection, we found no association between oedema and P-CRP and P-AGP, and the association between elevated acute phase reactants and clinical signs of infections was not very strong. Possibly, some reported symptoms were not caused by bacterial infections, or some children were not mounting a febrile response despite being infected.

### Household dietary diversity

We found that oedematous children came from households with a less diverse diet. For nuts and fresh fruits we found significant negative associations with oedema, while marginally significant associations were seen with fish, dairy products and green leafy vegetables. Since a sick child’s recent diet may be considerably different from the diet that lead to malnutrition, and recalling the pre-morbid diet is likely to be unreliable, we considered that household diet would give the most adequate picture of the child’s dietary environment. The lower dietary diversity in households of oedematous children was apparently not explained by higher degree of food insecurity.

A few other studies have investigated diets in relation to oedematous malnutrition, and remarkably few specific dietary risk factors have been identified [21,28,31]. The idea that oedematous malnutrition was caused by dietary protein deficiency was challenged when studies from India and Nigeria found no difference in dietary protein between children with oedematous and non-oedematous malnutrition [23,32]. One study, using an indirect method similar to ours, found that siblings to children with oedematous malnutrition consumed less tomato and eggs [31]. In contrast, a prospective study among at-risk children did not identify any single food item protecting against oedematous malnutrition, neither was the dietary diversity lower in those who developed oedematous malnutrition. However, in their DDS, foods in three of the seven food groups were virtually never consumed by the population, and while our score was based on diet during two weeks, theirs was based on diet consumed in one day [28]. While using food consumed during one day may be more specific when the diet is generally diverse, it is possible that comparing diet consumed over a longer period is more informative in populations consuming a very monotonous diet. Although we are not able to suggest any specific nutrient or food item that is lacking in the diet of children with oedematous malnutrition, our results suggest that it may indeed be worthwhile to examine the pre-morbid diet of these children, and that total dietary diversity, or the opposite, diet monotony, may be important for development of oedematous malnutrition.

### Birth weight and stunting

We found no association between recalled birth weight and oedema. Accordingly, our data contradict a recent report of higher birth weights in children with oedematous malnutrition [12]. With birth weight data available for only 88 we may have had insufficient power to detect a difference; however, we did not even see a trend for higher birth-weights in oedematous children. A reason for the discrepancy could be that the other study included children with non-oedematous SAM based on weight-for-age, which will inherently include more low-birth-weight children, while we included based on weight-for-height z-score and MUAC, less likely to select low-birth-weight children. However, our data should be interpreted with caution, being based on maternal recall; more than half reported a weight where the first decimal was 0 or 5 (e.g. 3.0 kg or 2.5 kg), indicating that their recall was imprecise.

Although children with the two types of malnutrition had similar HAZ, the non-oedematous were most stunted, adjusting for age. Children in our cohort, and elsewhere [33], tend to get progressively stunted with age; this explains how oedematous children with similar HAZ were less stunted, relative to their older age. An interpretation of this could be that oedematous children had been malnourished for a shorter time, or that non-oedematous children had been affected by more infections, which is known to cause stunting.

There are several limitations to our study: First, the observational and cross-sectional design precludes us from drawing conclusions about causality. However, by identifying associations we may formulate hypotheses, and later, in prospective studies test whether non-breastfed children, and children from households with low dietary diversity are indeed more likely to develop oedematous malnutrition. Second, some of the indicators used, like the DDS and the measure of appetite, were developed for the study, and have not been previously validated. Third, the exploratory nature of our study means that we make a considerable number of statistical tests. With the cut-off chosen for significance (p < 0.05) some of our findings are likely caused by chance. Since we did not adjust for multiple testing, our finding should be confirmed in other cohorts. On the other hand, some non-significant associations could be due to low power, with only 120 children included in our study.

## Conclusion

Breastfeeding, dietary diversity and infections were negatively associated with oedema in hospitalised children with SAM. Whether these factors play a causal role in the development of specific types of malnutrition remain unknown. Prospective studies among at-risk children are recommended to determine whether the factors are present before malnutrition develops, and whether the associations are also present in children in the community, not recruited from a hospital setting. It is possible, that clinical signs of infections may indicate different conditions than acute phase reactants, which should also be addressed in future studies.

## Additional file

Additional file 1: Figure S1. Dietary Diversity Questionnaire used in data collection.

## Abbreviations

AGP: α-1 acid glycoprotein
CI: 95% confidence interval
CRP: C-reactive protein
DDS: Dietary diversity score
HAZ: Height-for-age z-score
HIV: Human immunodeficiency virus
HFIAS: Household food insecurity access scale
MUAC: Mid-upper-arm circumference
OR: Odds ratio
SAM: Severe acute malnutrition
WHO: World Health Organization
