# Supplementary material for: Social, dietary and clinical correlates of oedema in children with severe acute malnutrition: a cross-sectional study
Source: BMC Pediatr. 2015 Mar 22;15:25. doi: 10.1186/s12887-015-0341-8 (PMC4383214; doi:10.1186/s12887-015-0341-8)
Supplement: Additional file 1: Figure S1. — Dietary Diversity Questionnaire used in data collection. [file 12887_2015_341_MOESM1_ESM.pdf]

V.Foodfrequency	Date:	Interviewer:	Patient ID
**During the last 2 weeks was the following**	**A: -served in the Household?**	**B: -if yes, eaten by nn?**	**C: If, no, would these foods have been served to nn before gettig sick?**
(1) Fish	(0)No (1)Yes no. of days:________ (8) Don't know	(0)No (1)Yes no. of days:________ (8) Don't know	(0)No (1)Yes (8) Don't know
(2) Nuts	(0)No (1)Yes no. of days:________ (8) Don't know	(0)No (1)Yes no. of days:________ (8) Don't know	(0)No (1)Yes (8) Don't know
(3) Eggs	(0)No (1)Yes no. of days:________ (8) Don't know	(0)No (1)Yes no. of days:________ (8) Don't know	(0)No (1)Yes (8) Don't know
(4) Meat	(0)No (1)Yes no. of days:________ (8) Don't know	(0)No (1)Yes no. of days:________ (8) Don't know	(0)No (1)Yes (8) Don't know
(5) Diary	(0)No (1)Yes no. of days:________ (8) Don't know	(0)No (1)Yes no. of days:________ (8) Don't know	(0)No (1)Yes (8) Don't know
(6) Green Vegetables	(0)No (1)Yes no. of days:________ (8) Don't know	(0)No (1)Yes no. of days:________ (8) Don't know	(0)No (1)Yes (8) Don't know
(7) Fresh fruit	(0)No (1)Yes no. of days:________ (8) Don't know	(0)No (1)Yes no. of days:________ (8) Don't know	(0)No (1)Yes (9) Don't know
